# High [CO_2_] and Temperature Increase Resistance to Cyhalofop-Butyl in Multiple-Resistant *Echinochloa colona*

**DOI:** 10.3389/fpls.2019.00529

**Published:** 2019-05-08

**Authors:** João Paulo Refatti, Luis Antonio de Avila, Edinalvo Rabaioli Camargo, Lewis Hans Ziska, Claudia Oliveira, Reiofeli Salas-Perez, Christopher Edward Rouse, Nilda Roma-Burgos

**Affiliations:** ^1^Department of Plant Protection, Federal University of Pelotas (UFPel), Pelotas, Brazil; ^2^United States Department of Agriculture - Agricultural Research Service, Beltsville, MD, United States; ^3^Department of Crop, Soil and Environmental Sciences, University of Arkansas, Fayetteville, AR, United States

**Keywords:** carbon dioxide, climate change, *Echinochloa*, junglerice, cyhalofop-butyl, multiple-resistance, resistance evolution

## Abstract

Changes in the environment, specifically rising temperature and increasing atmospheric carbon dioxide concentration [CO_2_], can alter the growth and physiology of weedy plants. These changes could alter herbicide efficacy, crop-weed interaction, and weed management. The objectives of this research were to quantify the effects of increased atmospheric [CO_2_] and temperature on absorption, translocation and efficacy of cyhalofop-butyl on multiple-resistant (MR) and susceptible (S) *Echinochloa colona* genotypes. *E. colona*, or junglerice, is a troublesome weed in rice and in agronomic and horticultural crops worldwide. Cyhalofop-butyl is a grass herbicide that selectively controls *Echinochloa* spp. in rice. Maximum ^14^C-cyhalofop-butyl absorption occurred at 120 h after herbicide treatment (HAT) with >97% of cyhalofop-butyl retained in the treated leaf regardless of [CO_2_], temperature, or genotype. Neither temperature nor [CO_2_] affected herbicide absorption into the leaf. The translocation of herbicide was slightly reduced in the MR plants vs. S plants either under elevated [CO_2_] or high temperature. Although plants grown under high [CO_2_] or high temperature were taller than those in ambient conditions, neither high [CO_2_] nor high temperature reduced the herbicide efficacy on susceptible plants. However, herbicide efficacy was reduced on MR plants grown under high [CO_2_] or high temperature about 50% compared to MR plants at ambient conditions. High [CO_2_] and high temperature increased the resistance level of MR *E. colona* to cyhalofop-butyl. To mitigate rapid resistance evolution under a changing climate, weed management practitioners must implement measures to reduce the herbicide selection pressure. These measures include reduction of weed population size through reduction of the soil seedbank, ensuring complete control of current infestations with multiple herbicide modes of action in mixture and in sequence, augmenting herbicides with mechanical control where possible, rotation with weed-competitive crops, use of weed-competitive cultivars, use of weed-suppressive cover crops, and other practices recommended for integrated weed management.

## Introduction

Climatic projections by the Intergovernmental Panel on Climate Change (IPCC) indicate an increase in global mean temperature (2.6–4.8°C) and CO_2_ atmospheric concentration ([CO_2_]) (730–1000 μmol⋅mol^-1^) by the end of the 21^st^ century (Gianessi, 2013; IPCC, 2014; Varanasi et al., 2015). The rapidity of these changes is likely to have consequences on a number of human activities, including agriculture.

Physical environmental changes, such as precipitation and temperature can alter agronomic practices including land preparation, planting, irrigation, and fertilizer application. However, biological interactions, particularly pest population dynamics and severity, may also be affected. Weeds, as an example, can cause 100% crop loss when not controlled. The degree of loss varies by crop, cultivar, weed species, weed infestation level, location, year, and farming practices (Abouziena and Haggag, 2016; Soltani et al., 2016). In corn alone, yield losses due to weeds ranged from 10 to 83% in North America and averaged 51% in Eastern Canada between 2007 and 2013 (Soltani et al., 2016). Rice yield losses from competition with *Echinochloa* spp. across the globe range from 10 to 79% (Smith, 1974, 1988; Hill et al., 1985; Stauber et al., 1991; Fischer et al., 1997; Chin, 2001). Overall, increasing [CO_2_] and temperature may alter dominant weed species and increase weed problems (Ziska and Dukes, 2011).

Herbicides are the primary tools used to control weeds and minimize economic losses in crop production. Herbicide use has been increasing globally in traditionally low-herbicide-using countries, including China, India and parts of Africa where hand labor is becoming increasingly scarce and expensive (Gianessi, 2013). However, changes in [CO_2_] and temperature can potentially alter herbicide efficacy (Varanasi et al., 2015; Korres et al., 2016). For example, stimulation of weed growth by elevated [CO_2_] could reduce time in the seedling stage when weeds are most sensitive to herbicides. Alternatively, [CO_2_]-induced changes in stomatal conductance could also reduce herbicide absorption. Increased temperatures, or temperature extremes, could increase herbicide efficacy by accelerating absorption and translocation of foliar herbicides; but could also induce rapid metabolism, which reduces herbicide efficacy in target plants (Johnson and Young, 2002). In addition, increased temperature and [CO_2_] can change the leaf surface characteristics by increasing leaf thickness, or changing the viscosity of the cuticle wax, with subsequent reductions in herbicide absorption (Ziska and Bunce, 2006).

Among the negative consequences of increasing temperature and [CO_2_] could be the acceleration of weed resistance evolution to herbicides, specifically, non-target-site resistance. Resistance to herbicides is an example of evolved avoidance mechanism to abiotic stressors. Stress avoidance mechanisms in plants are oftentimes similar, or common, among various environmental stress factors. Non-target-site-based resistance (NTSR) mechanisms, which include increased herbicide metabolism, herbicide sequestration, reduced absorption and translocation increased protection from strong oxidants, and overproduction of herbicide target (Powles and Yu, 2010; Délye, 2013), are strongly influenced by changes in climate (see Ramesh et al., 2017). Non-target site resistance can trigger multiple resistance to herbicides or endow resistance to herbicides not yet used on the weed population. Cases of multiple resistance and NTSR are increasing (Roma-Burgos et al., 2019). For example, we observe increasing cases of NTSR to acetyl-CoA carboxylase (ACCase) inhibitors, in addition to the already widespread target-site resistance to these herbicides (Heap, 2019). Strong NTSR to ACCase inhibitors in grasses has been attributed primarily to herbicide detoxification aided by increased production of monooxygenases (cytochrome P450s), which facilitate phase I detoxification reactions and increased activity of different classes of glutathione-S-transferase (GST) enzymes, which facilitate phase II detoxification (Powles and Yu, 2010; Cummins et al., 2013; Délye, 2013).

Junglerice [*Echinochloa colona* (L.) Link], together with barnyardgrass [*E. crus-galli* (L.) Beauv.], is very difficult to control in rice as it mimics rice at the vegetative stage (Peerzada et al., 2016). Cyhalofop-butyl and fenoxaprop-P-ethyl are ACCase inhibitors currently used in lowland rice fields for postemergence control of *Echinochloa* species and other grass weeds (Scott et al., 2018). These herbicides are absorbed by the leaves and are translocated primarily via the phloem to the meristematic tissues where the herbicide inhibits lipid synthesis. The depletion of lipids stops the growth of cells, organs, and tissues, leading to plant death. The repeated use of grass herbicides (as with most other herbicides) has resulted in the evolution of resistance to ACCase inhibitors among some *Echinochloa* populations (Huan et al., 2013; Heap, 2014; Rouse et al., 2018a). Resistance to ACCase inhibitors are almost equally driven by mutations at various loci involved in herbicide binding at the target site and by various NTSR mechanisms (Délye et al., 2011; Kaundun, 2014). Selective grass herbicides (i.e., diclofop-methyl, tralkoxydim, pinoxaden) are inactivated in plants through hydroxylation, by cytochrome P450s in phase I detoxification reactions (Wenger et al., 2012) and subsequent conjugation by GTs or GSTs in phase II reactions (Milner et al., 2001; Brazier et al., 2002). Thus, metabolic resistance is a common NTSR mechanism, along with some cases of reduced absorption or translocation (De Prado et al., 2005).

Whether enhanced [CO_2_] and/or temperature can alter the efficacy of selective grass herbicides, and whether there is differential response between susceptible and resistant grass genotypes to these factors are not known. The objective of the current research was to quantify the effects of increased atmospheric [CO_2_] and temperature on the absorption, translocation, and efficacy of cyhalofop-butyl on multiple-resistant and susceptible *E. colona* genotypes under simulated climate change conditions.

## Materials and Methods

Experiments were conducted in 2016 and 2017 at the Altheimer Laboratory in the Department of Crop, Soil and Environmental Sciences, University of Arkansas, Fayetteville, United States.

### Experiment 1. Effect of [CO_2_] on the Response of Cyhalofop-Resistant and -Susceptible *E. colona* to Cyhalofop-Butyl

Five seedlings of *E. colona* were grown in 0.2 L pots filled with soil (Dardanelle silt loam: silty, mixed, active, thermic Typic Udifluvents) in four replications. The pots were placed in trays and kept in a growth chamber maintained at 23/35°C night/day temperature. The trays were filled with water, allowing irrigation by capillary movement. Three days before herbicide treatment (HAT), nitrogen fertilizer was applied at the recommended field use rate for irrigated rice. To determine the effect of CO_2_ levels on plant growth and response to herbicide, three factors were tested as a factorial treatment structure. Factor A was CO_2_ concentration: ambient (*a*[CO_2_]) at 400 ± 50 μmol⋅mol^-1^ and elevated (*e*[CO_2_]) at 700 ± 50 μmol⋅mol^-1^. These values were chosen based on the predicted [CO_2_] at the end of this century (IPCC, 2014). To control [CO_2_] levels, a system was designed and built, and installed in two plant growth chambers. This system centralizes information in a single processor, thus facilitating control of [CO_2_] and recording of data. The processor was programmed to collect sensor readings every 10 s. This interval was determined from previous tests to achieve the best stability of the desired [CO_2_]. Data from each CO_2_ sensor were stored in the processor internal memory for subsequent calculation of the amount of CO_2_ to add into the growth chambers intermittently. The CO_2_ levels were maintained by automated comparison of the desired concentration programmed into the processor with the CO_2_ sensor reading. To satisfy the prescribed conditions, the quantity of CO_2_ to be injected into the chamber was calculated using a mathematical model established in previous experiments (unpublished data).

Factor B was genotype of *E. colona*, characterized in previous experiment as susceptible (S) and multiple-resistant (MR) to propanil and quinclorac with low level of resistance to cyhalofop (Rouse and Burgos, 2017; Roma-Burgos et al., 2018). This population was collected from East Arkansas, in a field historically planted with rice and occasionally rotated with soybean. Cyhalofop was rarely used in this field. Factor C was cyhalofop-butyl treatment (with, or without). The experimental units (pots) were arranged in a completely randomized design. At the three-leaf stage, the plants were sprayed with commercial formulation of cyhalofop-butyl (Clincher^®^, Dow AgroSciences, United States) at 1.09 L⋅ha^-1^ in a laboratory spray chamber equipped with flat-fan nozzles (TeeJet 80015; Spraying Systems Co., Wheaton, IL, United States) at 290 kPa pressure, delivering 183 L⋅ha^-1^. Crop oil concentrate (Agri-Dex; Helena Chemical Company, Collierville, TN, United States) was added to the herbicide mixture at a final concentration of 1% v/v. Visible injury, plant height, and shoot biomass were evaluated 1 week after HAT. The experiment was repeated.

### Experiment 2. Effect of [CO_2_] on Absorption and Translocation of Cyhalofop-Butyl in *E. colona*

The same *Echinochloa* genotypes were planted and cultured as described above. Plants were harvested at 6, 12, 24, 72, and 120 h after HAT. Commercial (or “cold”) cyhalofop-butyl was applied to seedlings as described above. Immediately after the cold HAT, the plants were moved to the radioisotope laboratory.

A stock solution of technical-grade ^14^C-labeled cyhalofop-butyl (specific activity = 32.8 mCi/mM) was prepared in acetonitrile (500 μL) and water (2:1 by vol.) solution. An aliquot of the non-radiolabeled cyhalofop-butyl (Clincher^®^, 1.09 L⋅ha^-1^, Dow AgroSciences, United States) spray solution was spiked with an aliquot of ^14^C-labeled cyhalofop-butyl stock solution to prepare spotting solutions with a specific activity of 0.25 kBq⋅μL^-1^. Four 1 μL droplets of this spotting solution were applied on the adaxial surface of the second fully expanded leaf, for a total of 1 kBq⋅μL^-1^ per plant. Once the droplets had dried, the pots were transferred to growth chambers set for each CO_2_ concentration.

The treated leaves were harvested at designated times and placed in 20 mL glass vials containing 3 mL methanol. Each vial was shaken gently by hand for 10 s to remove the non-absorbed herbicide. The rinsate was mixed with 10 mL of scintillation cocktail (Ultima Gold^TM^; PerkinElmer Inc., Waltham, MA, United States) and the radioactivity was quantified using a liquid scintillation spectrometer (LSS) (Packard Tri-Carb 2100TR liquid scintillation spectrometer; Packard Instrument Corp., Downers Grove, IL, United States). Absorption of ^14^C-cyhalofop-butyl was calculated by subtracting the amount of radioactivity in the leaf rinsate from the total applied. At each harvest time, the plants were fractioned into treated leaf (TL), leaf above TL, leaf below TL, and roots. For statistical analysis, factors A and B were [CO_2_] and genotype, respectively. Factor C was harvest time and factor D was plant sections. After sectioning, the plant tissues were dried at 60°C for 48 h and then oxidized (OX500^TM^; R. J. Harvey Instrument Corp., Tappan, NY, United States). The ^14^CO_2_ evolved during tissue combustion was trapped in a vial containing 15 mL of scintillation cocktail (Carbon-14 Cocktail; R. J. Harvey Instrument Corp.) and quantified using LSS, as mentioned above. The experiment was repeated.

At each harvest time, one whole plant was used to visualize herbicide translocation using autoradiography. The roots were rinsed with tap water and non-absorbed ^14^C-labeled cyhalofop-butyl was removed by rinsing the treated leaf with methanol as described above. The plants were pressed immediately with absorptive paper, and leaves were spread carefully to prevent the treated leaf from contacting other plant tissues. The plants were then stored at -55°C for 48 h, exposed to a phosphorscreen for 48 h and then scanned in a Storm 820 PhosphorImager^TM^ (Molecular Dynamics Inc., Sunnyvale, CA, United States).

### Experiment 3: Response of *E. colona* to Cyhalofop-Butyl at Different Temperatures

The same methodology described in Experiment 1 was used, except that Factor A was temperature: 23/35°C (night/day) and 26/38°C, simulating the current actual (*a*T) and predicted average temperature (*e*T) at the end of this century, respectively (Varanasi et al., 2015). Visible injury, plant height, and shoot biomass were evaluated 1 week after HAT. The experiment was repeated.

#### Statistical Analysis

The treatment effects were the same in both runs of the experiment; therefore, the two runs were combined in the analysis. Data were tested for normality and homogeneity of variance, transformed when needed, and subjected to three-way analysis of variance. The interaction among treatment factors were tested. When significant differences were detected, Tukey’s test (*P* < 0.05) was performed to separate treatment means. The *F*-test (*P* < 0.05) was used to compare two means. Statistical analysis was performed using R Software (Foundation for Statistical Computing, Vienna, Austria). Figures were prepared using Sigmaplot v. 12.5 for Windows (Systat Software, Inc., San Jose, CA, United States).

## Results and Discussion

Three-way interactions among factors wasere not significant in ANOVA, for this reason; therefore, the results were presented separately in two-way factor interactions are presented.

### Effect of High [CO_2_] on the Efficacy of Cyhalofop-Butyl on *E. colona*

**FIGURE 1:**
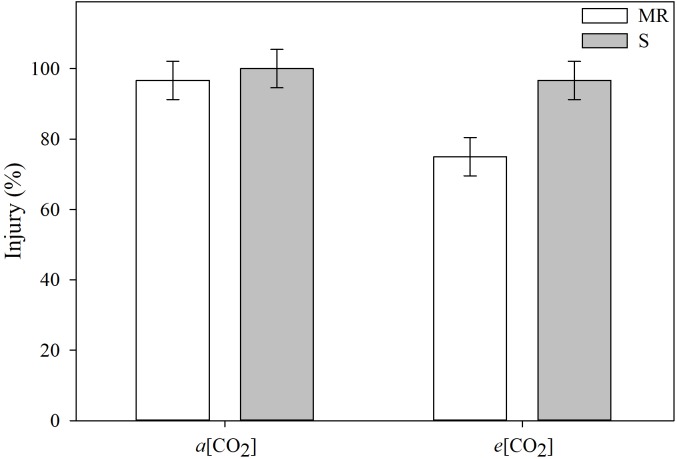
Injury of *Echinochloa colona* genotypes, 7 days after treatment, grouped by CO_2_ concentration. MR, multiple-resistant to propanil and quinclorac with low resistance to cyhalofop; S, susceptible; *a*[CO_2_] = 400 ± 50 μmol⋅mol^-1^; *e*[CO_2_] = 700 ± 50 μmol⋅mol^-1^. *F*-test (*P* < 0.05); Fayetteville, AR, United States.

[CO_2_] differentially affected the extent of *E. colona* injury between MR and S genotypes from cyhalofop-butyl treatment (Figure 1). At ambient [CO_2_], the *E. colona* genotypes responded, similarly, to cyhalofop-butyl; the S genotype was killed and the MR genotype was nearly killed 7 days after treatment. Under elevated [CO_2_], cyhalofop-butyl did not kill the S plants 100%, but the reduction in herbicide activity was not significant. Conversely, the herbicide efficacy on MR plants declined significantly from almost 100% at *a*[CO_2_] to <80% at *e*[CO_2_]. This level of weed control would be considered a failure under field conditions by crop producers as 90% control is the minimum level deemed excellent (Scott et al., 2018).

The basis for increased resistance of MR-*E. colona* to cyhalofop-butyl under *e*[CO_2_] is not fully understood. Thus far, we know that this MR population is highly resistant to quinclorac and propanil via increased herbicide detoxification, concomitant with constitutive upregulation of activity in carbon fixation, fatty acid synthesis, and trehalose biosynthesis pathways (Rouse and Burgos, 2017; Roma-Burgos et al., 2018; Rouse et al., 2018b). Transcriptome data and subsequent validation of relative expression of candidate genes indicate a joint action of cytochrome P450s, UGT glycosyltransferase, GSTs, and key genes in the trehalose biosynthesis pathway to effect not only herbicide detoxification, but also increased protection from abiotic stress (Roma-Burgos et al., 2018; Rouse et al., 2018b). This population has not been selected intensively with ACCase herbicides in the field; yet the lipid synthesis pathway is constitutively upregulated. Thus, we hypothesize that the low-level resistance to cyhalofop-butyl is a latent effect of mechanisms that endow high levels of resistance to quinclorac and propanil. With the constitutive upregulation of carbon assimilation, lipid biosynthesis, and abiotic-stress-protection genes, the potential exists for elevated tolerance to other herbicides such as ACCase inhibitors. It has been reported recently that resistance to ACCase inhibitors in large crabgrass (*Digitaria sanguinalis* L.) is due to overexpression of ACCase (Laforest et al., 2017). Hence, we propose that *e*[CO_2_] provides more carbon resources to fuel the upregulated carbon assimilation machinery in MR-*E. colona*, producing more ingredients for fatty acid synthesis, ACCase enzyme, or protection proteins, which could dilute the effect of fatty-acid-synthesis-inhibitors. Late watergrass [*E. phyllopogon* (Stapf) Koso-Pol.], a close relative and also a major weed problem in rice, has evolved resistance to cyhalofop-butyl via increased metabolism of cyhalofop acid, coupled with reduced herbicide absorption through the cuticle (Ruiz-Santaella et al., 2006a).

Other studies have shown reduced herbicide efficacy under *e*[CO_2_] for C3 and C4 weedy species (Ziska and Teasdale, 2000; Ziska et al., 2004; Manea et al., 2011). For example, glyphosate is less effective on *Eragrostis curvula* (Schrad.) Nees and *Paspalum dilatatum* Poir under *e*[CO_2_] (Manea et al., 2011). These studies suggested that the reduction in glyphosate efficacy could be related to [CO_2_]-induced increases in plant size (biomass and leaf area), resulting in herbicide dilution. Such growth stimulation, however, contradicts the general idea that C4 plants *per se*, do not benefit from rising [CO_2_]. While additional information on this point is desired, there are previous reports that C4 weeds could respond more to elevated [CO_2_] than C4 crops (Ziska and Bunce, 1997).

### Effect of [CO_2_] on Absorption of ^14^C-Cyhalofop-Butyl in *E. colona*

**FIGURE 2:**
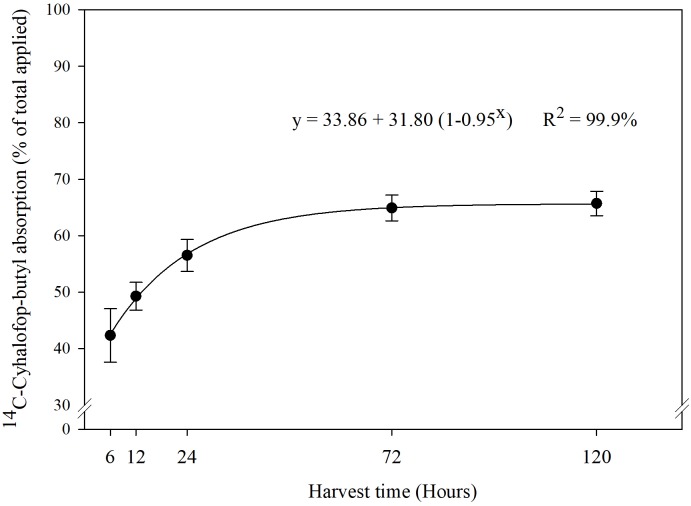
Percentage of ^14^C-cyhalofop-butyl absorbed, of the total applied on *Echinochloa colona*, at 6, 12, 24, 72, and 120 h (95% confidence intervals). Commercial formulation sprayed at V3 stage with 1% crop oil concentrate and spotted with 1 kBq⋅μL^-1^; Fayetteville, AR, United States.

Increased [CO_2_] can induce morphological, physiological, and anatomical changes in plants that could affect herbicide absorption and translocation rate (Manea et al., 2011). Previous research indicated that plants (C3 and C4) grown under elevated [CO_2_] have thicker cuticle and increased leaf pubescence (Ainsworth and Long, 2005). These traits could reduce herbicide entry into plant leaves. Besides increasing leaf thickness, *e*[CO_2_] can also induce partial stomatal closure, which may reduce the absorption and efficacy of herbicides (Jackson et al., 2011). Stomatal closure results in reduced stomatal conductance, which in turn results in reduced transpiration flow, eventually resulting in reduced herbicide absorption, especially those applied on the soil (Bunce and Ziska, 2000; Ziska and McClung, 2008). However, in our experiment, the absorption of ^14^C-cyhalofop-butyl into leaves of *Echinochloa* seedlings did not decline under *e*[CO_2_]. The interaction effects of [CO_2_], genotype, and sampling time on absorption of ^14^C-cyhalofop-butyl were not significant (*P* > 0.05). Herbicide absorption was almost 45% at 6 HAT and reached a maximum of 65% at 120 HAT (Figure 2), averaged across genotypes and [CO_2_]. Herbicide absorption did not differ between MR- and S genotypes and was not affected by [CO_2_] (Supplemental Figure 1). Differential tolerance to cyhalop-butyl in grasses is attributed partly to differential absorption into leaves owing to differences in cuticular traits. Rice, for example, has an even coating of epicuticular waxes, which limits the absorption of cyhalofop-butyl to only 30% in 24 h after treatment vs. 77% in the susceptible *E. oryzoides*, which has an uneven coating of waxes (Ruiz-Santaella et al., 2006b). Anatomical modification of leaf surfaces toward reduction of herbicide absorption requires long-term evolutionary adaptation; such change cannot be expected in this study.

### Effect of [CO_2_] on ^14^C-Cyhalofop-Butyl Translocation in *E. colona*

**FIGURE 3:**
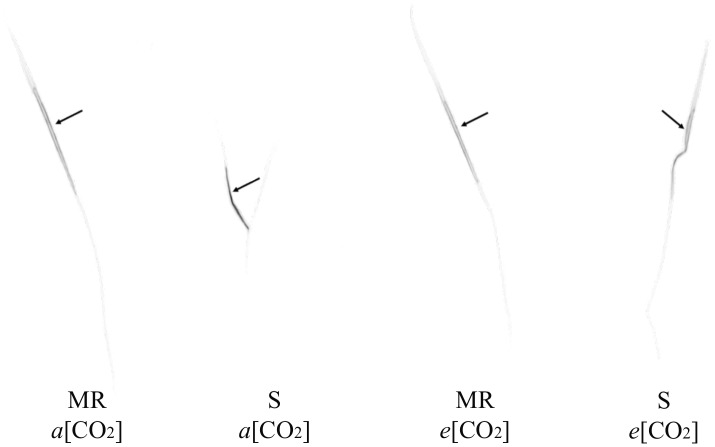
Autoradiography of ^14^C-cyhalofop-butyl in MR and S *Echinochloa colona* genotypes, cultivated in *a*[CO_2_] and *e*[CO_2_] at 120 HAT. *a*[CO_2_] = 400 ± 50 μmol⋅mol^-1^; *e*[CO_2_] = 700 ± 50 μmol⋅mol^-1^; MR, multiple-resistant to propanil and quinclorac with low resistance to cyhalofop-butyl; S, susceptibl;. Fayetteville, AR, United States.

Aryloxyphenoxypropanoate herbicides, such as cyhalofop-butyl, are formulated as esters to facilitate absorption into leaves, but are converted to their acid (herbicidal) form by esterases such as what happens with cyhalofop-butyl in rice (*Oryza sativa* L.) and in its major grass weed, *Echinochloa* spp. (Ruiz-Santaella et al., 2006b). The herbicide is translocated in its acid form in the plant. Therefore, translocation refers to cyhalofop acid. The interaction effect between [CO_2_] and leaf tissue on detected radioactivity was significant (Table 1). This indicated differential herbicide distribution in the plant between *a*[CO_2_] and *e*[CO_2_], averaged across genotypes. The translocation of cyhalofop within the plant also differed between genotypes, averaged across [CO_2_]. Most of the absorbed herbicide (>98%) remained in the treated leaf, regardless of the genotype and [CO_2_] (Table 1 and Figure 3). Slightly more herbicide moved out of the treated leaf at *e*[CO_2_] than *a*[CO_2_], but these minute amounts could not be detected by phosphorimaging. The small amount that was translocated was distributed slightly differently under *a*[CO_2_] vs. *e*[CO_2_]. These small differences in herbicide translocation, though significant in some plant sections, did not matter as the efficacy of cyhalofop declined at *e*[CO_2_]. The slight increase in herbicide translocation under *e*[CO_2_] may be associated with increased translocation of nutrients and increased metabolic activity because of higher available CO_2_. Cyhalofop is known to have limited mobility within the plant. In *E. phyllopogon*, 98% of applied ^14^C-cyhalofop remained in the treated leaves at 12–24 HAT, regardless of the resistance phenotype (Ruiz-Santaella et al., 2006a).

**Table 1:** ^14^C-cyhalofop-butyl translocation to various plant tissues, under two CO_2_ concentrations ([CO_2_]) and two *Echinochloa colona* genotypes, 120 h after treatment.

Plant sections	^14^C-cyhalofop-butyl (% of the total ^14^C absorbed)							
	
	[CO_2_]				Genotypes			
		
	*a*[CO_2_]		*e*[CO_2_]		MR		S	
Above treated leaf	0.36	Abc	0.29	Ac	0.28	Ab	0.37	Ac
Below treated leaf	0.48	Ab	0.59	Ab	0.43	Bb	0.65	Ab
Root	0.29	Ac	0.31	Ac	0.30	Ab	0.31	Ac
Treated leaf	98.83	Aa	98.65	Ba	98.90	Aa	98.58	Ba


*Means followed by the same lowercase letters within a column are not different based on Tukey’s test (P < 0.05). Means followed by the same uppercase letters within a row are not different based on Tukey’s test (P < 0.05). a[CO_*2*_] = 400 ± 50 μmol⋅mol^*-1*^; e[CO_*2*_] = 700 ± 50 μmol⋅mol^*-1*^; MR, multiple-resistant to propanil and quinclorac with low resistance to cyhalofop-butyl; S, susceptible; Fayetteville, AR, United States.*

Averaged across [CO_2_] levels, the MR genotype retained more ^14^C-cyhalofop in the treated leaf than the S genotype (Table 1). Again, although significant, these numbers are very close (98.9% vs. 98.6%). This showed slightly more translocation in the S genotype than in the MR genotype. This also indicates that the mechanism lending low-level resistance to cyhalofop partly involves minimal reduction in translocation, at least under *a*[CO_2_] condition. This minor difference in cyhalofop translocation between genotypes does not explain the large reduction in cyhalofop efficacy on the MR genotype under *e*[CO_2_].

Under ambient conditions, other researchers (Fernández et al., 2016) reported no differences in the translocation of ^14^C-diclofop, another selective grass herbicide in the same chemical family as cyhalofop, among herbicide-resistant and -susceptible *Cynosurus echinatus*. The translocation of cyhalofop also did not differ among resistant and susceptible *E. phyllopogon* (Ruiz-Santaella et al., 2006a). Such cases indicate the involvement of other resistance mechanisms, perhaps increased metabolism of the herbicide or mutation at the ACCase binding site.

### Effect of High Temperature on the Efficacy of Cyhalofop-Butyl on *E. colona*

**FIGURE 4:**
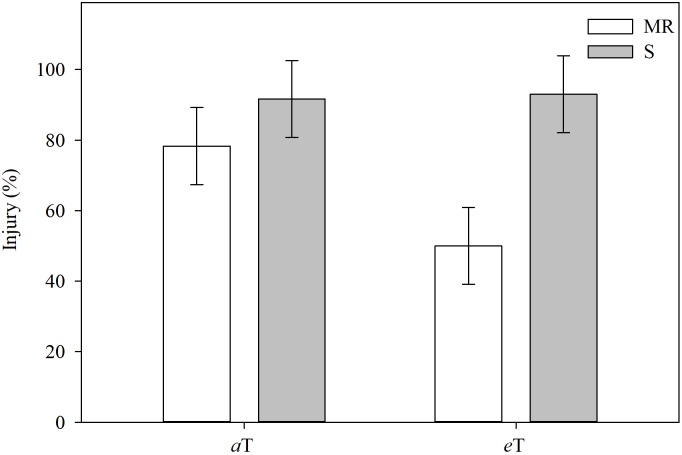
Injury in different *Echinochloa colona* genotypes, 7 d after treatment, grouped by temperature levels. MR, multiple-resistant to propanil and quinclorac with low resistance to cyhalofop; S, susceptible; *a*T = 23/35°C (night/day); *e*T = 26/38°C (night/day). *F*-test (*P* < 0.05); Fayetteville, AR, United States.

As with [CO_2_], the interaction effect of temperature and *E. colona* genotypes on cyhalofop-butyl efficacy was significant (Figure 4). Under ambient temperature, the activity of cyhalofop-butyl on S plants was numerically higher (about 90%) than on MR plants (about 80%) at 7 DAT. At elevated temperature, the efficacy of cyhalofop-butyl on the S genotype remained high, but the efficacy on the MR genotype declined significantly to about 50%. Temperature influences several processes related to herbicide efficacy. These include the rate of water absorption and transpiration, the rate of leaf development, cuticle thickness, and stomatal number and aperture (Bailey, 2004; Rodenburg et al., 2011). The ability of plants to tolerate (minimize or sustain damage and recover) the negative effects of abiotic stress, including herbicides, improves with increase in temperature up to the optimum point for growth and development. Thus, we observe, for example, increased injury in rice from bispyribac-sodium herbicide applied during a cold period (Martini et al., 2015). Even evolved resistance to herbicides, such as that of johnsongrass (*Sorghum halepense* L.) and rigid ryegrass (*Lolium rigidum* L.) to glyphosate could be overcome under cold temperature (Vila-Aiub et al., 2013). This paradigm does not apply to all weed-herbicide-temperature interactions, of course. Plant response is modified by the herbicide mode of action and other mitigating factors such as the extent to which low temperature impedes herbicide absorption and translocation, or herbicide activation in the plant (as with proherbicides), resulting in reduced efficacy at low temperatures.

Reduced efficacy at high temperature generally may be due to increased metabolism of, or increased protection from, the herbicide as a consequence of maximal physiological conditions at high temperature. There is support for this hypothesis such as the findings of Godar et al. (2015) where the activity of mesotrione on Palmer amaranth declined when the temperature increased from 25 to 40°C. The same was reported by Matzrafi et al. (2016) on the reduction in activity of pinoxaden (an ACCase inhibitor) on *Brachypodium hybridum* and other grasses under high temperature. These researchers found significantly higher levels of the glucose-conjugated metabolite of pinoxaden herbicide in *B. hybridum* under high temperature compared to low temperature. In the same study, the authors also observed that pinoxaden-resistant *B. hybridum* plants metabolized pinoxaden faster at elevated temperature.

In the case of *E. colona* in the current study, high temperature increased resistance to cyhalofop-butyl in the MR genotype by a significant margin, whereas herbicide efficacy remained similar in the S genotype. This data is highly informative for achieving sustainable weed management in a warming climate. How is increased resistance possible under high temperature? The MR genotype shows constitutive upregulation of key genes implicated in alleviation of abiotic stress and detoxification of xenobiotics (Roma-Burgos et al., 2018; Rouse et al., 2018b). The MR genotype also shows hypersensitive response to propanil, which allows quick death of affected cells and faster recovery (i.e., regrowth) when coupled with other constitutive coping mechanisms (Roma-Burgos et al., 2018). All together, we propose that these mechanisms are enhanced under high temperature, which is favorable for *Echinochloa* growth, resulting in increased resistance to cyhalofop-butyl. Follow-up research is needed on the specific effect of rising temperature on these metabolic interactions and the consequences on weed response to herbicides.

### Effect of High Temperature on Absorption of ^14^C-Cyhalofop-Butyl in *E. colona*

**FIGURE 5:**
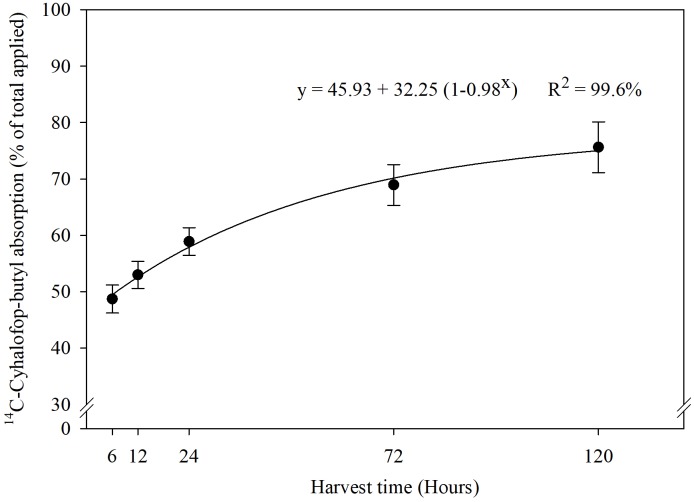
^14^C-cyhalofop-butyl absorption by *Echinochloa colona* at different times after application (95% confidence intervals). Commercial formulation was sprayed at V3 stage with 1% crop oil concentrate and the leaf was spotted with 1 kBq⋅μL^-1^; Fayetteville, AR, United States.

Absorption of ^14^C-cyhalofop-butyl in *E. colona* under different temperatures (Figure 5) followed the same pattern as obtained with different [CO_2_] levels. The interaction effects of temperature, genotype, and sampling time on absorption of ^14^C-cyhalofop-butyl were not significant (*P* < 0.05). Herbicide absorption approached a plateau at 120 HAT with maximum absorption at 75% averaged across genotypes and temperatures.

### Effect of High Temperature on ^14^C-Cyhalofop-Butyl Translocation in *E. colona*

**FIGURE 6:**
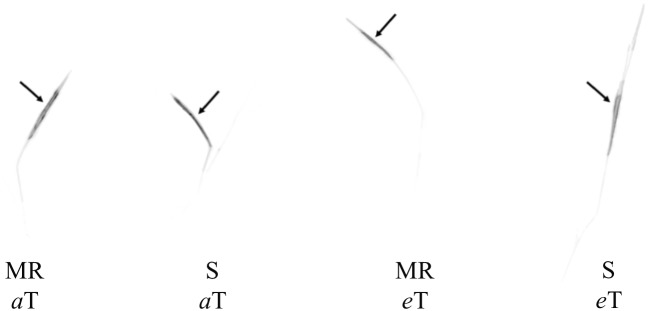
Autoradiography of ^14^C-cyhalofop-butyl in MR and S *Echinochloa colona* genotypes, grown in *a*T and *e*T, at 120 HAT. MR, multiple-resistant to propanil and quinclorac with low resistance to cyhalofop-butyl; S, susceptible; T = 23/35 °C (night/day); *e*T = 26/38 °C (night/day); Fayetteville, AR, United States.

The interaction effect of temperature and genotype on the translocation of ^14^C-cyhalofop-butyl was not significant (Table 2). A large proportion (at least 97.86%) of absorbed ^14^C-cyhalofop-butyl remained in the treated leaf of both genotypes (Table 2 and Figure 6). More herbicide remained in the treated leaf at elevated temperature (98.6%) averaged across genotypes, indicating reduced translocation. Although small, this difference was significant. This minimal reduction in translocation might have contributed to the reduced herbicide efficacy at high temperature across genotypes, but does not explain the large reduction in herbicide efficacy on the MR genotype compared to that of the S genotype. Possible reasons for increased resistance to cyhalofop-butyl under high temperature are discussed previously. In related research, Godar et al. (2015) also observed reduced translocation of mesotrione in Palmer amaranth (*Amaranthus palmeri* S. Watson) grown at 40°C compared to plants grown at 25°C. Optimal temperature is expected to promote optimal translocation of herbicide, resulting in higher herbicide efficacy. However, the effect of temperature on the metabolic activity of the plant overrides increased herbicide translocation, as herbicide detoxification also increases at high temperature (Martini et al., 2015; Matzrafi et al., 2016).

**Table 2:** ^14^C-cyhalofop-butyl translocation in two *Echinochloa colona* genotypes, multiple-resistant (MR) and susceptible (S), averaged over ambient (*a*T), and elevated (*e*T) temperature, 120 h after treatment (HAT).

Plant sections	^14^C-cyhalofop-butyl in each plant section at 120 HAT (% of the total absorbed)							
	
	*a*T		*e*T		MR		S	
Above treated leaf	0.99	Ab	0.72	Bb	0.77	Bb	0.95	Ab
Below treated leaf	0.62	Ac	0.32	Bc	0.44	Ac	0.50	Ac
Root	0.53	Ac	0.36	Bc	0.53	Ac	0.36	Bc
Treated leaf	97.86	Ba	98.59	Aa	98.26	Aa	98.19	Aa


*Means followed by the same lowercase letters within a column are not different based on Tukey’s test (P < 0.05). Means followed by the same uppercase letters within a row are not different based on Tukey’s test (P < 0.05). aT = 23/35°C (night/day); eT = 26/38°C (night/day); MR, multiple-resistant to propanil and quinclorac with low resistance to cyhalofop-butyl; S, susceptible; Fayetteville, AR, United States.*

### Effect of [CO_2_] and Temperature on *E. colona* Biomass Production

**FIGURE 7:**
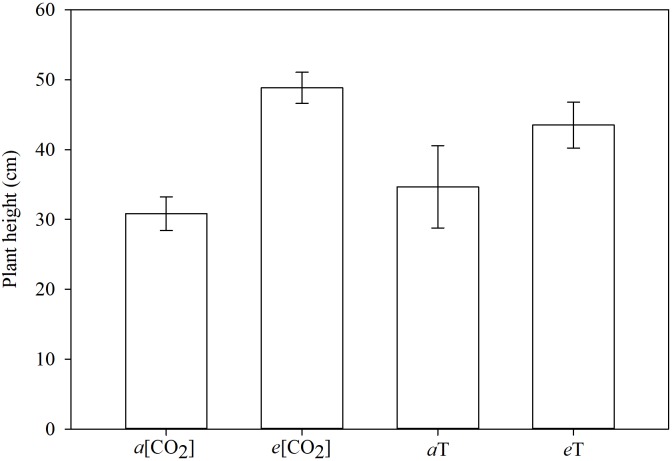
Plant height, 21 days after planting, grouped by environmental conditions. Means followed by the same letter do not differ statistically based on Tukey’s test (*P* < 0.05). *a*[CO_2_] = 400 ± 50 μmol⋅mol^-1^; *e*[CO_2_] = 700 ± 50 μmol⋅mol^-1^; *a*T = 23/35°C (night/day); *e*T = 26/38°C (night/day). *F*-test (*P* < 0.05); Fayetteville, AR, United States.

**FIGURE 8:**
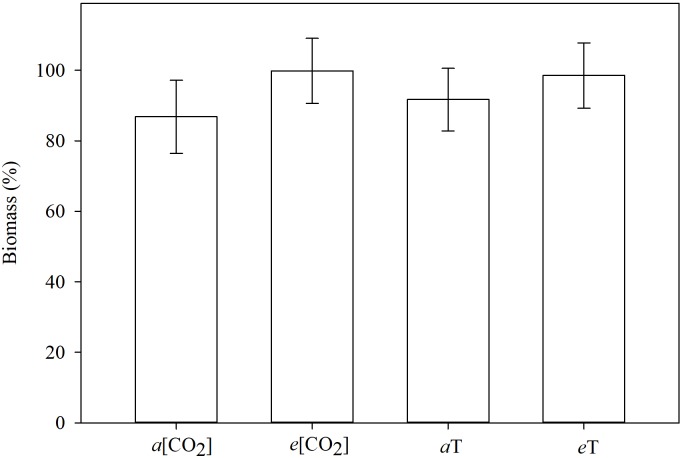
Shoot biomass (%), relative to the non-treated check, 21 days after planting, grouped by environmental conditions. Means followed by the same letter do not differ statistically based on Tukey’s test (*P* < 0.05). *a*[CO_2_] = 400 ± 50 μmol⋅mol^-1^; *e*[CO_2_] = 700 ± 50 μmol⋅mol^-1^; *a*T = 23/35 °C (night/day); *e*T = 26/38°C (night/day). *F*-test (*P* < 0.05); Fayetteville, AR, United States.

The tallest plants were those grown in elevated CO_2_ concentration or high temperature (Figure 7). The MR and S plants did not differ in height within each of these conditions. Averaged across genotypes, taller plants under climate-change conditions did produce numerically more biomass than the shorter plants under ambient conditions, but could not be separated statistically in this regard (Figure 8). This just reflects the fact that in grasses, culm or leaf elongation does not necessarily equate to more biomass, up to a point. The height difference of the main culm between environmental conditions was only within 20 cm. Unless this was accompanied with significantly more tillering (which it was not), the corresponding increase in biomass would be of lesser magnitude as our data portrayed. Temperature and [CO_2_] are major determinants of plant growth and development. Concurrent increases in temperature and [CO_2_] are expected to have significant effects on plants, but some interaction effects are turning out to be contrary to expectations. The herbicide degradation rate or metabolism could be faster in big plants than in small ones, and big plants need higher doses for the herbicide to reach all target sites at effective levels. Thus, it takes more herbicide to kill big plants than small ones as indicated by formulation labels for foliar herbicides specifying small seedlings, or recommending high rates on bigger plants and even more for non-selective desiccation of fully grown vegetation. It is apparent that climate changes will influence (directly or indirectly) how weeds are managed.

The shoot biomass did not differ between *E. colona* genotypes nor between environmental conditions (data not shown). The plants were small when harvested (3 weeks after planting), only 7 days after HAT. The observed differences in levels of injury were not reflected on the amounts of biomass.

On a side note, *E. colona* germinated faster, regardless of genotype, under high temperature (Supplemental Table 1). The germination capacity was the same (90%) at ambient and high temperature. Under ambient temperature maximum germination occurred 6 days after seeding. Seeds attained 50% of maximum germination 1–1.5 days earlier under high temperature than in ambient temperature. This conforms with the germination behavior of *E. colona* (Chauhan and Johnson, 2009). Optimum germination occurs between 30 and 35°C, but it can germinate up to 40°C. Tropical weeds, such as *E. colona*, germinate faster at higher temperature. Therefore, under elevated temperature, we can expect *E. colona* to get established faster and compete with the crop more vigorously early in the season, intensifying the need for effective weed management from crop planting to harvest. *E. colona* is a C4 plant (Giussani et al., 2001); it responds more to high temperature than the C3 crop, rice.

The data collectively indicate that increasing temperature or CO_2_ levels alter the nature of plant-herbicide interaction, eventually resulting in reduced efficacy of cyhalofop-butyl, increased resistance of MR *E. colona* to ACCase inhibitors, and increased potential competitive ability with the crop. Elevated [CO_2_] and high temperature, two fundamental aspects of global climate change, could act as factors for resistance evolution to herbicides. As mentioned earlier, this MR population harbors NTSR mechanisms to quinclorac (hormone-type action) and propanil (PS II inhibitor). Since the general mechanisms underlying NTSR [herbicide detoxification or sequestration, reduced translocation, protection from damage, overproduction of herbicide target (Délye, 2013)] are the same across weedy species, and the drivers of these mechanisms are affected by abiotic stresses, climate change can accelerate the evolution of NTSR to herbicides of different modes of action in weeds. In nature, atmospheric [CO_2_] and temperature are going to change concurrently as climate changes. Our follow-up research will focus on modeling the interaction effect of increasing [CO_2_] and temperature on the response of some key weedy species to herbicides. The expectation is that C3 crops (i.e., rice, soybean) would approach the photosynthetic capacity of C4 weeds (i.e., *Echinochloa* spp., *Amaranthus* spp.) under excess supply of CO_2_. Photosynthetic capacity is also expected to increase further when CO_2_ and temperature are not limiting. However, weeds generally respond more to resource changes than crops and many major weeds today, like many crops, are C3 (i.e., *Abutilon theophrasti* L., *Ambrosia* spp., *Avena fatua* L., *Chenopodium album* L., *Ipomoea* spp.) (Ziska and Bunce, 1997; Ziska and McClung, 2008; Ziska and Dukes, 2011). Many troublesome weeds in rice production, for example, are C3 plants; the most notorious of these is its close relative, weedy rice (*Oryza sativa* L.) (Delouche et al., 2007). Although both are of the same genus and species, weedy rice responds more to CO_2_ enrichment (Ziska and McClung, 2008) and nitrogen addition (Burgos et al., 2006). It is also troubling that cases of resistance, or multiple resistance, to herbicides are increasing among key rice weeds, as in other crops (Roma-Burgos et al., 2019). Our data, and those of several other research groups, show that high atmospheric [CO_2_] or high temperature reduces sensitivity of weed species to various herbicides. More research is needed to understand the complex effects of climate change (combined effect of high temperature and high CO_2_) on crop-weed-herbicide interactions to help agriculture practitioners attain and sustain food and fiber security in the face of changing climate.

To mitigate rapid resistance evolution under a changing climate, weed management practitioners must implement measures to reduce the herbicide selection pressure. These measures include reduction of weed population size through reduction of the soil seedbank, ensuring complete control of current infestations with multiple herbicide modes of action in mixture and in sequence, augmenting herbicides with mechanical control where possible, rotation with weed-competitive crops, use of weed-competitive cultivars, use of weed-suppressive cover crops, and other practices recommended for integrated weed management (Norsworthy et al., 2012). Implementing the most effective combination of practices to reduce weed infestation is complex, time-consuming, and costly. Currently, weed resistance problems are evolving in developed, developing, or underdeveloped countries wherever herbicides are used intensively (Heap, 2019). Farmers in “resource-sufficient” regions do not hold an advantage over those in “resource-poor” regions. Therefore, it will require a drastic change in mentality, across all groups of people influencing or conducting weed management, to manage weeds with the goal of averting resistance rather than to fix resistance problems.

## Conclusion

The absorption of cyhalofop-butyl in herbicide-susceptible and multiple-resistant *E. colona* does not change under elevated [CO_2_] or high temperature. The translocation of cyhalofop out of the treated leaf is modified slightly by increased [CO_2_] or temperature, but the major effect is the significant increase in resistance to cyhalofop-butyl in the multiple-resistant genotype. Therefore, elevated [CO_2_] or high temperature increases the resistance level of *E. colona* to the ACCase-inhibitor, cyhalofop-butyl. Differential response between S and MR genotypes under high [CO_2_] or temperature would, in turn, accelerate the evolution of herbicide resistance in response to global climate change. The fact that the MR genotype has extreme resistance to quinclorac and propanil, which involves NTSR mechanisms as shown in background studies, results of this current study have broad implications on the impact of climate change on the evolution of NTSR to herbicides in weeds.

## Author Contributions

JR, NR-B, LdA, and EC designed the experiments. JR, CO, and RS-P performed the experiments. JR analyzed the data. JR and NR-B wrote the manuscript. NR-B, JR, CR, LZ, and LdA discussed the results and their implications and revised the manuscript.

## Conflict of Interest Statement

The authors declare that the research was conducted in the absence of any commercial or financial relationships that could be construed as a potential conflict of interest.

## Supplementary Material

Table 1 *Echinochloa colona* germination under ambient (*a*T) and elevated (*e*T) temperature, averaged across genotypes, Fayetteville, AR, United States.

Figure 1 Absorption of ^14^C-cyhalofop-butyl in susceptible- (S) and multiple-resistant (MR) *Echinochloa colona* under ambient and elevated [CO_2_] 120 h after application. Lines represent standard errors of the mean. Commercial formulation was sprayed at V3 stage with 1% crop oil concentrate and the leaf was spotted with 1 kBq⋅μL^-1^; Fayetteville, AR, United States.
