# Supplementary material for: High [CO2] and Temperature Increase Resistance to Cyhalofop-Butyl in Multiple-Resistant Echinochloa colona
Source: Front Plant Sci. 2019 May 8;10:529. doi: 10.3389/fpls.2019.00529 (PMC6518978; doi:10.3389/fpls.2019.00529)
Supplement: Table 1 — Echinochloa colona germination under ambient (aT) and elevated (eT) temperature, averaged across genotypes, Fayetteville, AR, United States. [file Table_1.docx]

**Supplemental Table 1.** *Echinochloa colona* germination under ambient (*a*T) and elevated (*e*T) temperature, averaged across genotypes, Fayetteville, AR, USA.

| Days | Germination (%)^1^ | | | | |
| --- | --- | --- | --- | --- | --- |
|  | *a*T | |  | *e*T | |
| 2 | 0 | Ad |  | 0 | Ae |
| 3 | 5 | Bd |  | 20 | Ad |
| 4 | 20 | Bc |  | 60 | Ac |
| 5 | 50 | Bb |  | 75 | Ab |
| 6 | 90 | Aa |  | 90 | Aa |

^1^Ten seeds were planted per pot, with four replications, and two runs. Means followed by the same lowercase letter within a column are not different based on Tukey's test (*P* < 0.05). Means followed by the same uppercase letter within a row are not different based on Tukey's test (*P* < 0.05). *a*T = 23/35 °C (night/day); *e*T = 26/38 °C (night/day); Fayetteville, AR, USA.
