# Supplementary figures and images for: High [CO2] and Temperature Increase Resistance to Cyhalofop-Butyl in Multiple-Resistant Echinochloa colona
Source: Front Plant Sci. 2019 May 8;10:529. doi: 10.3389/fpls.2019.00529 (PMC6518978; doi:10.3389/fpls.2019.00529)

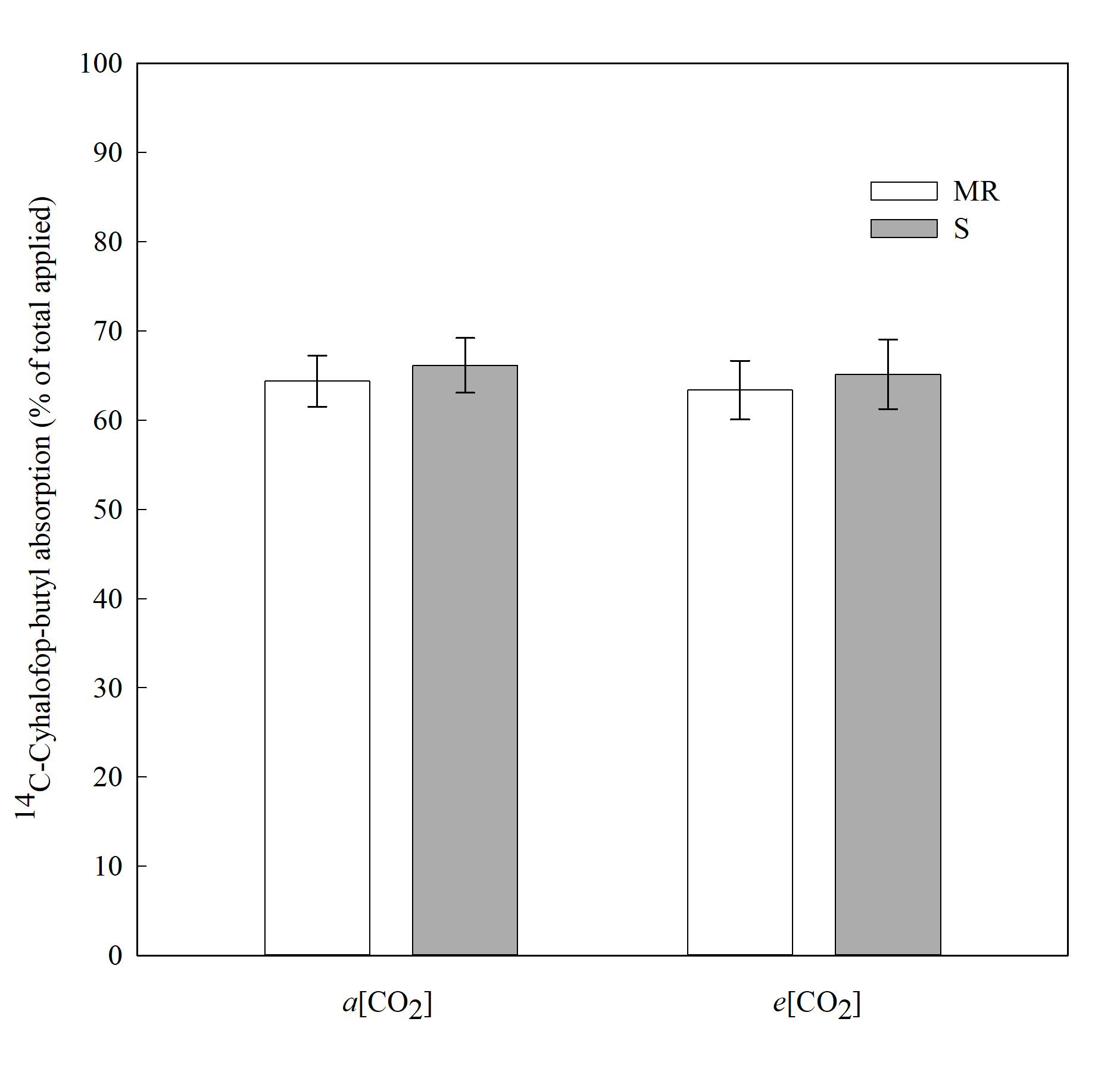

Supplement: Figure 1 — Absorption of 14C-cyhalofop-butyl in susceptible- (S) and multiple-resistant (MR) Echinochloa colona under ambient and elevated [CO2] 120 h after application. Lines represent standard errors of the mean. Commercial formulation was sprayed at V3 stage with 1% crop oil concentrate and the leaf was spotted with 1 kBq⋅μL-1; Fayetteville, AR, United States. [file Image_1.jpg]
